# Anaesthesia for children with cancer

**DOI:** 10.1016/j.bjae.2024.03.007

**Published:** 2024-05-09

**Authors:** Z. Kostense, J. de Ruiter

**Affiliations:** University Medical Centre, Groningen, The Netherlands

**Keywords:** adjuvant chemotherapy, cancer, children, paediatric anaesthesia

**Learning objectives**

By reading this article, you should be able to:

• Describe the impact of cancer and cancer treatment in children with cancer presenting for anaesthesia.
• Recall the adverse effects of cancer therapy.
• Discuss the complexity and challenges of anaesthesia delivery in children with cancer.

**Key points**

• Childhood cancer can be present at any age with varying signs and symptoms.
• The impact on children with cancer can be related to direct effects of the tumour, and the effects of treatment.
• The adverse effects of chemotherapy and radiotherapy can have both long- and short- term detrimental effects.
• It is common for children with cancer to require anaesthesia at different stages from the beginning of their cancer diagnosis, during treatment and follow-up.
• The delivery of anaesthesia in children with cancer can be conducted in operating theatre and non-operating theatre settings.

Over the last five decades there have been advances in services for childhood cancer with an overall improvement in survival rates and outcomes. One of the key aspects to the success is the acknowledgement of the need for a structured and standardised good practice within childhood cancer. Many initiatives have been set up to improve outcomes in children with cancer through research, clinical trials and pooling of observational childhood cancer data, including local oncological grassroots organisations such as the UK Children's Cancer Study Group formed in 1977.^1^^,^^2^ This is one of many programmes that led to collaborations between similar groups, nationally and globally. Despite an increase in cancer survival, the survival outcomes remain low in lower- and middle-income countries.^2^ In response to the discrepancies of health inequalities in childhood cancer, the WHO has set up the CureAll framework as a global blueprint for children with cancer.

The drive for improvement in childhood cancer provision has inherently increased the need for anaesthetic services. Many anaesthetists would have encountered children with cancer or childhood cancer survivors at least once in their practices. Some of the children have outlived their cancer and go into their adulthood cancer-free. Some of whom may carry the lasting impact of their cancer and cancer therapy. The complexity of childhood cancer and its treatment, deserve full attention to ensure good and safe anaesthetic care.

## Childhood cancer statistics

Despite the diversity of childhood cancer, the likelihood of encountering a certain type of cancer depends on age. Unlike in adult cancer, most of the cancer cells derive from embryonal tissues that have acquired mutation.^3^ The peak incidence of childhood cancer types vary according to their age groups (Table 1). The commonest childhood cancers across all ages are haematological (30%), central nervous system (CNS) tumours (26%) and lymphoma (11%).^2^^,^^4^^,^^5^

**Table 1:** Common cancer presentation according to age groups globally. There may be some discrepancies in the data for children with cancer above 15 yrs old between higher-income countries and middle- and lower-income countries.^2^^,^^4^^,^^5^

Top three childhood cancers according to age group	Children age group (yrs)			
	0–4	5–10	11–14	15–19
1	Leukaemia	Leukaemia	Leukaemia	Lymphoma
2	CNS tumour	CNS tumour	CNS tumour	Epithelial tumour and melanoma
3	Embryonal tumour	Lymphoma	Lymphoma	Leukaemia

## Associated pathology and challenges in childhood cancer

Though anaesthesia is rarely a rate-limiting step for the care of children with cancer it may be challenging or high risk. This can be secondary to cancer-related pathology such as the presence of mediastinal mass in the child or the adverse effects of cancer therapy. The common problems that may be encountered in delivering anaesthesia to children with cancer are described below.

## Cancer-related problems

### Mediastinal mass

The commonest site for a chest mass in childhood cancer is the mediastinum.^6^, ^7^, ^8^ Initial presentation may be an incidental finding of a local mass without any symptomatic problems. There is a risk of anaesthetic complications such as respiratory and cardiovascular problems because of the proximity of the airway, heart and great vessels. Compression, or direct involvement of nearby anatomical structures, can be manifested as cough, stridor, dyspnoea, orthopnoea, superior vena cava syndrome and Horner syndrome.^6^^,^^7^ Most children with Hodgkin lymphoma, non-Hodgkin lymphoma and acute lymphocytic leukaemia (ALL) have mediastinal involvement at diagnosis. The anaesthetic complications resulting from anterior mediastinal masses in children have been discussed in detail in this journal.^7^

### Raised intracranial pressure

Increased intracranial pressure (ICP) can result in low Glasgow Coma Scale (GCS) score. Primary brain tumours are the most common childhood intracranial tumours. Brain metastases are far less common in children. Increased ICP may present acutely or develop insidiously. The presentation may be secondary to obstruction to the flow of cerebrospinal fluid, infection, bleeding or mass effect from the tumour.^8^ Common signs and symptoms include changes in vision, nausea and vomiting. A younger child may be inconsolable, restless and irritable. An older child may complain of headache.

### Deranged electrolytes

Electrolyte imbalances can be primary or secondary causes of the cancer presentation. Tumour lysis syndrome (TLS) is an important primary cause, and is considered an oncological emergency.^9^^,^^10^ The massive lysis of lymphoblastic cells releases intracellular metabolites such as potassium and metabolites of purine and protein into the systemic circulation in large amounts. This can result in hyperkalaemia, hyperuricaemia, hyperphosphatemia and secondary hypocalcaemia. The rapid electrolyte derangement, if uncorrected, leads to cardiac arrythmias, seizures and renal impairment and is associated with 21% mortality.^10^ Tumour lysis syndrome can occur spontaneously before cancer treatment and is commonly seen in ALL. Rarely, TLS may occur after hormonal treatment and irradiation therapy. There have been several case reports of TLS developing after receiving dexamethasone as antiemetic.^10^^,^^11^ Therefore, the use of dexamethasone as standard antiemetic in children with cancer is not advisable.^11^ Secondary causes of deranged electrolytes are hormonal deregulation, dehydration and sepsis. Syndrome of inappropriate antidiuretic hormone secretion (SIADH) is an example of hormonal deregulation that causes hyponatraemia. Any primary brain cancer and lymphoma can cause SIADH leading to seizures and encephalopathy.

### Haematological disorders

#### Coagulopathy

There is a general increased risk of thrombosis and bleeding complications in children with cancer because of the prothrombotic nature of cancer. Cytokines, procoagulant and fibrinolytic proteins are released by cancer cells. Some patients are asymptomatic and coagulopathy is only detected when specific clotting tests are carried out. Other causes of coagulopathy include the usage of procoagulant medication, long-term access catheters, chemotherapy, radiotherapy and surgery.^12^ The standard indications for transfusion are applicable in children with cancer although certain products such as irradiated blood are reserved for children with Hodgkin lymphoma. We refer to paediatric oncology transfusion guidelines for further details.^13^

#### Thrombocytopenia

Tumour infiltration in the bone marrow and platelet sequestration in the spleen can cause thrombocytopenia commonly seen in children with acute myeloid leukaemia (AML) and ALL. A platelet count below 10×10 L^−1^ is associated with spontaneous bleeding.^12^^,^^13^^,^^14^ The potential complications are high where there is an urgent need for platelet transfusion before surgery or lumbar puncture. Recent guidelines suggest a platelet concentration >40×10^9^ L^−1^ for lumbar puncture before initiating platelet transfusion.^15^

#### Neutropenia

Neutropenia is defined when the neutrophil count is <1.5×10^9^ L^−1^.^12^^,^^16^ This is commonly seen in ALL and AML where the normal bone marrow function is replaced by abnormal maturation and dysregulated proliferative immature cells. The lower the neutrophil count, the greater the susceptibility of the children to developing infection. Symptoms suggestive of neutropenia include fever, pain and chills.^12^^,^^16^ Patients with neutropenia are also susceptible to recurrent infections with varying rates of morbidity and mortality. A combination of fever and neutropenia is considered a medical emergency where wide-spectrum antibiotics are indicated. Although neutropenia can be the first presentation of cancer, it is more common after chemotherapy. Some patients with neutropenia are asymptomatic and detected only through their differential white blood cell count.^16^

#### Anaemia

More than 90% of leukaemia and lymphoma patients have anaemia.^14^, ^17^^,^^18^^,^^19^ Anaemia is a common symptom in children with bone cancer, neuroblastoma and brain tumour.^19^ The symptoms can be subtle and can go undetected. Common presentations are general malaise, tachypnoea, heart murmur and dizziness. In the younger age group, there would be poor feeding and failure to thrive. The threshold of treating anaemia varies but it is common to treat anaemia when the haemoglobin is <8 g dl^−1^.^14^, ^18^, ^19^, ^20^

## Cancer treatment problems

Cancer treatment depends on the tumour and the cancer stages. A multimodal approach is often used where a combination of chemotherapy, radiation therapy and surgery including transplant is proposed.^21^ Immunotherapy has been gaining a role in cancer therapy over the last decade and is considered in certain cancer types.^21^, ^22^ Cancer treatments are not without risks and can have both short- and long-term consequences.^21^^,^^23^, ^24^, ^26^, ^27^

## Chemotherapy

Chemotherapy drugs are designed to be cytotoxic to rapidly dividing cells by various mechanisms.^21^, ^28^ Chemotherapeutic drugs eradicate the cancer cells but can also destroy normal cells. Adverse effects of chemotherapy are mostly temporary. However, some long-term effects can occur.

### *Cardiomyopathy*

Anthracyclines such as doxorubicin are the most cardiotoxic class of chemotherapeutic drugs.^21^, ^25^, ^28^ Cyclophosphamide, used in treating lymphoma, is also cardiotoxic. The predisposing factors for toxicity include dosing schedule and cumulative doses. Acute toxicity can occur within days from initiating treatment and are generally reversible. Chronic toxicity can occur with a progressive decline in cardiac function; symptoms include congestive heart failure.^21^, ^24^, ^28^ Cardiomyopathy can develop within months to years after treatment. The surveillance and follow-up care are dependent on the patient's condition, local resources and availability of specialist teams. This has prompted a call for proper guidance. As a response, the European Society of Cardiology has produced standardised guidelines on cardio-oncology for patients with cancer.^26^

### *Anorexia and malnutrition*

Loss of appetite is prevalent in those receiving chemotherapy.^8^, ^21^ Chemotherapy-induced mucositis, nausea, vomiting, diarrhoea or constipation may cause anorexia and perpetuate the psychological fear of eating. Often there is an altered smell or taste which may discourage children from eating. Malnutrition, poor weight gain and weight loss have a great impact in terms of children's growth, development and well-being. Poor nutritional status in children has the potential to delay or decrease cancer treatment and magnify treatment-related toxicity.^29^ Enteral or parenteral feeding is recommended to prevent further deterioration.

### *Risk of infection and immunosuppression*

The cytotoxic nature of the chemotherapy affects the immunological component of the bone marrow. Therefore, all children with cancer started on chemotherapy are immunosuppressed^8^^,^^21^, ^28^, ^30^ During the treatment phase, they are vulnerable to infection. The patients are closely monitored and started on supportive treatment when early signs of infection are detected. These infectious agents are often opportunistic organisms which may begin as colonisation. Each hospital admission and every medical intervention carries a significant risk of developing infection.

## Radiotherapy

Radiation therapy uses a high-energy radiation beam to destroy cancer cells and shrink tumours.^8^^,^^21^, ^27^ This leads to cell death of both cancer cells and normal tissues by causing damage to cellular DNA. Radiation may be external or internal, proton or photon. It is painless and radiation machines are used to target a designated specific cancer area in the body using a measured dose, spread in series of fractions over days or weeks.^21^ Proton beam therapy requires a lower radiation dose and is more precise compared with photon beam therapy (See Table 2).^29^, ^31^ This is especially important in children as radiation therapy increases the susceptibility to developing secondary malignancy in later life.^7^, ^33^ Most of the adverse effects of radiation are temporary. However, some may have long-term effects.^26^^,^^27^^,^^29^, ^31^, ^32^, ^33^

**Table 2:** Differences between proton and photon radiation.

Photon	Proton
Uses ionising photon (X-rays)	Uses positive charged particle (hydrogen)
Produced with a linear accelerator	Produced with accelerators known as cyclotrons and synchrotrons
The beam loses energy but does not stop, so a dose is delivered behind the target	The beam stops at a specific depth based on the energy, so there is no exit dose
The machine can be housed in a standard hospital building	The machine has to be placed in a specifically designed self-contained building

### *Skin, mucosa changes and xerostomia*

The acute response to radiation causes desquamation of dry and moist areas and presents as mucositis, pruritis, ulcers and pain. Trismus may develop between 3 and 6 months and longer after radiation therapy.^33^ Severe mucositis in the mouth, head and neck causes severe pain.^26^^,^^27^ Xerostomia or dry mouth occurs when the salivary gland is damaged secondary to radiation in the head and neck region causing a reduction in saliva production. Analgesia, dietary changes and meticulous oral care and in some children parenteral nutrition are advised as part of the management.^21^^,^^27^

### *Growth and cognitive function*

Radiation therapy has inhibitory effects on the growth of the developing bones. This can impair bone growth production, for example, asymmetrical bony protuberance on the face. Other organs such as the thyroid, endocrine and pituitary glands can also be affected. Children with cancer treated with radiation therapy may develop precocious puberty and hypothyroidism. Of those who received craniospinal radiation, between 50% and 80% experienced growth failure. Behavioural changes, IQ decline and cognitive development problems such as memory problems are known consequences of radiation therapy.^21^, ^27^, ^29^

### *Cardiopulmonary effects*

There is a dose-response relationship between chest-directed radiation and the development of late cardiopulmonary diseases. Cardiopulmonary effects include pericarditis, arrhythmia and fibrosis. In both partial and full lung radiation therapy, impaired pulmonary function is common.^8^, ^21^^,^^27^

### Immunotherapy

Immunotherapy is a form of treatment that is designed to use the patient's own immune system to eradicate cancer cells.^21^, ^22^, ^23^ Several therapeutic approaches are being studied, including cytokines, manipulation of T cells and vaccines. In adult malignancies, immunotherapy has shown promising results, but the field is still in its infancy and especially true for childhood cancers.^21^ Immunotherapies are generally thought to have fewer long-term toxicities than chemotherapy and radiation. Short-term adverse effects are very common such as fever and headache. More serious adverse effects can also occur such as myalgia, autoimmune reactions and neurotoxicity.

## Anaesthesia

Children with cancer undergo multiple investigations, surgical procedures and interventions that require general anaesthesia (GA) as part of their management. The reasons for anaesthetists' involvement are to reduce distress and trauma in children and offer comfort and analgesia.^34^ For some investigations or therapies, children are unable to lay still or cooperate during such sessions because of their age, pain, fear and lack of understanding. The contact with anaesthesia may be frequent and intermittent. As the child progressed from diagnoses to treatment, contact with anaesthetic services may become more frequent. Requests for anaesthesia in these patients are rarely declined. The urgency of diagnosis and treatment requires anaesthetists to facilitate medical and surgical management decisions in a timely manner to prevent disease progression.

The delivery of anaesthesia in children with cancer is not only limited to the operating theatre. Often, other procedures requiring anaesthesia are conducted outside the operating theatre. Non-operating room anaesthesia occurs in remote areas such as radiology and endoscopy units. Non-operating room anaesthesia is associated with higher risks than anaesthesia in the operating theatre supported by recent literature reviews.^35^^,^^36^ The unfamiliarity with the remote environment, the variability of equipment and monitoring modalities can lead to increased incidences of adverse events.^8^^,^^35^^,^^36^ Detailed planning and a fully trained team in delivering anaesthetic care in remote areas should be part of the standard operating procedure.

Common procedures where anaesthesia is required for a child with cancer are described below. Many children are often anxious and scared by the experience. We also discuss the psychosocial impact of cancer on children and their family.

### Diagnostic investigations

The initial contact with anaesthesia services may be in confirming the suspected cancer diagnosis. This includes investigations such as MRI or CT scan for oncological imaging or lumbar puncture for confirmation of ALL. Biopsies from suspicious lumps and bumps may be indicated and performed by the surgeon or radiologist. On initial presentation, the patients can be acutely ill or symptomatically well. As most of the patients would have their basic investigations done, it is pertinent that other symptoms and signs of cancer presentation including both positive and negative findings are actively looked for. Some of these may bear weight to the proposed procedure.^8^^,^^21^ For example, thrombocytopenia is relevant for a diagnostic lumbar puncture compared with a child requiring GA for an MRI investigation. However, the presence of a mediastinal mass would be relevant in both as it may influence how a safe GA can be delivered.

### Vascular access

Children with cancer often require long-term i.v. access. This is used for giving drugs and chemotherapy, hydration, blood sampling and i.v. access for anaesthesia. Long-term i.v. access reduces the frequent need for injections and blood sampling, and prevents vein damage and psychological trauma from needles being continually sited into the children.^8^ Vascular access insertion is performed by the general surgical or interventional radiology team. Before insertion, attention to the patients' coagulation status and platelet count is vital to prevent haematoma and bleeding.^13^, ^15^ For children with vascular access port device, local anaesthetic cream can be applied over the injection site to numb the area. This offers comfort when the needle is inserted into the port. Children with long-term intravascular access are also susceptible to line infection.^16^ Aseptic protocols must be adhered to strictly when the catheter is used to minimise the risks of infection. At the end of the treatment, the vascular access is removed, almost always under anaesthesia.

### Nutrition

Children require good nutrition to cope with their energy expenditure during rapid growth and development. Additional energy requirements in children with cancer are needed not only as part of the general development, but also to cope with the impact of cancer and cancer treatment. If these requirements are not met, the growth pattern will decline with consequences in the long term.^8^^,^^30^ Adequate nutrition is important to support the healing process from cancer therapy and dampen the effects of cancer toxicity. Therefore, nutrition is extremely important in children with cancer. When the child is unable to maintain adequate nutrition, it is common that their nutrition is supplemented with a nasogastric feeding tube. Such scenarios are seen in children who develop oral mucositis and loss of appetite during chemotherapy. Anaesthesia support may be required for insertion of a nasogastric tube or a gastrostomy. For those unable to have enteral nutrition, vascular access is required for parenteral nutrition.

### Surgery

The aim of surgical treatment is to remove as much of the tumour from the patient as possible. This can be a curative or palliative cancer therapy. Other surgical involvement includes vascular access insertion and diagnostics such as biopsy. The principles and conduct of anaesthesia in such surgeries do not differ from the standard anaesthetic practice. However, the factors discussed above should be taken into consideration when delivering anaesthesia.^7^^,^^8^^,^^34^^,^^35^

### Intrathecal chemotherapy

Intrathecal chemotherapy is performed in children with leukaemia, lymphoma and brain tumours.^21^ Children who received intrathecal chemotherapy can be acutely unwell at the start of the therapy. Depending on the centre, the treatment can be performed by nurse specialists, paediatricians or oncologists. Sedation or GA is given to tolerate the intermittent therapy. The procedure can be carried out in operating theatres or other locations. Intrathecal chemotherapy can be repeated several times over a few months up to several years. Intrathecal drug error can occur with fatal consequences through injection of an i.v. dose of the same intrathecal medication. Safety checks, proper drug labelling, equipment and personnel training are part of the local protocol to avoid such incidents.

### Radiotherapy

Radiotherapy treatments are usually performed remotely from the operating theatre. The anaesthetic team is unable to remain with the patient at the point when the radiation therapy is delivered. Here, distant monitoring is required. For proton therapy, the centres are usually housed in a separate building because of infrastructure and safety aspects of the proton radiation emission. The therapy session is repeated daily for up to 6 weeks in a radiotherapy centre. For the treatment to be effective, the sessions should be continuous. This is the key for radiotherapy to be successful.^31^ Therefore, postponing anaesthesia in the patient for radiotherapy should be avoided if at all possible. For children who are unable to lay still during radiation therapy, sedation or GA is required.^8^, ^32^ Their daily contact with anaesthesia would focus not only on the safety aspect of anaesthetic delivery, but also on the psychosocial aspect of the children.^34^^,^^37^, ^41^ As the therapy session is daily for a set period, a familiar routine which suits the child and family helps gain trust and reduce anxiety levels.

### Critical care support

Early contact with the paediatric intensive care unit is needed when there is a progressive neurological deterioration or GCS<13, or if a child with cancer is acutely septic and unwell. The anaesthetist must be prepared to manage the sick child and assist with emergency interventions such as tracheal intubation. This is often done together with the paediatric oncologist and paediatric intensivist. The sick child may need to proceed with the necessary diagnostics such as radiological imaging which may lead to emergency surgery. In some patients, interhospital transfer may be required to bring the patient to a specialist hospital for further management. The risks of anaesthetic adverse events are highest in this group where proper planning with critical care backup may be necessary.

### Acute pain

Around 70% of children with cancer suffer from acute pain and 49% of children reported pain on presentation.^34^^,^^37^^,^^38^ In some centres, there are dedicated paediatric oncological pain specialists including anaesthetists, paediatricians and psychologists. Patient- and nurse-controlled analgesia with opioids or ketamine are options for treating pain.^34^^,^^38^ The pain assessment is conducted based on the patient's cognitive level and their ability to communicate and self-report. The parents and the nursing staff can assist in reporting the pain for the patient.

### Follow-up scans, late effects of cancer treatment and surveillance

The trajectory of childhood cancer care does not end with the completion of cancer therapy. The latter stages include follow-ups, surveillance, monitoring effects of the cancer therapy and development of late cancers.^21^^,^^25^ Anaesthesia may be needed for follow-up and surveillance investigations such as MRI. The anaesthetic approach in this latter stage of cancer can differ as the child with cancer would have undergone and completed the cancer therapy. The child may have readjusted in their life after cancer, grown up and achieved their childhood milestones. Some of them may develop long-term adverse effects after their cancer therapy which can cause permanent physiological changes.^21^, ^25^^,^^27^^,^^29^ The late effect of cancer can be seen at the later stages of childhood and can continue further into their adulthood.

## Psychological impact on the child with cancer and their family

Cancer coping strategies, understanding of the illness and its impact on the child with cancer are highly dependent on the development and cognitive function of the child. For example, toddlers rely heavily on their parents, whereas adolescents may wish to assert their independence.^34^^,^^39^ As many children with cancer are often accompanied by their parents during anaesthesia, it is important to acknowledge their presence. Parents or carers and siblings of children with cancer play a vital supportive role to the child with cancer during and after the period of cancer treatment.^39^, ^41^ They see and deal with the suffering of their child, face their emotional upheaval and simultaneously continue dealing with their own lives with the rest of the family. Therefore, a child-oriented and sensitive approach is advocated during the delivery of anaesthesia.^8^^,^^34^^,^^40^ Parents should be allowed to be part of the process if they wish to do so. In older children with cancer, they would want to be part of the shared decision-making of their illness including how they wish to be induced. Children should be allowed to address their requests and needs. Where possible, their requests should be respected and adhered to. This would strengthen trust in patient–doctor relationships and reduce the impact of stress and trauma associated with anaesthesia and cancer.

It is important to realise that the distressing impact of cancer on both the child and their family goes beyond anaesthesia delivery. Psychosocial development issues such as behavioural problems and disruption to daily family routine, and a constant fear of death are some of the many issues faced by children with cancer and their families. Both child and family would need support to address the psychosocial distress and psychological impact during cancer treatment and beyond.^40^^,^^41^ A multidisciplinary approach is recommended where the patient and family are allowed to express their fears and needs. The support team is not only limited to medical and nursing specialists such as in oncology, paediatrics and anaesthesia, but includes allied health professionals such as play therapists, psychologists and social workers.^8^, ^41^ This approach allows comprehensive and cohesive care thus meeting the demands of the psychosocial needs of the child with cancer.

## Conclusions

The perioperative care for children with cancer depends on the stage of cancer, the condition of the child with cancer at that point in time and the type of procedure required. During the entire cancer trajectory, the patients' physiological condition may change accordingly. The psychosociological impact should not be underestimated and attention should be given to reduce the mental burden. The anaesthetist needs to be aware of the basic understanding and impact of cancer and cancer therapy to design a safe and sensible approach without compromising the patients' safety, emotional and physical well-being. Whether this is conducted in, or outside the operating theatre, the principles of anaesthesia remain the same taking into account the above factors.

## Declaration of interests

The authors declare that they have no conflicts of interest.

## MCQs

The associated MCQs (to support CME/CPD activity) will be accessible at www.bjaed.org/cme/home by subscribers to *BJA Education*.
